# Smart Driving Technology for Non-Invasive Detection of Age-Related Cognitive Decline

**DOI:** 10.3390/s24248062

**Published:** 2024-12-18

**Authors:** Peter Serhan, Shaun Victor, Oscar Osorio Perez, Kevin Abi Karam, Anthony Elghoul, Madison Ransdell, Firas Al-Hindawi, Yonas Geda, Geetika Chahal, Danielle Eagan, Teresa Wu, Francis Tsow, Erica Forzani

**Affiliations:** 1 School of Electrical, Computer and Energy Engineering, Arizona State University, Tempe, AZ 85281, USA; pserhan@asu.edu; 2 Center for Bioelectronics and Biosensors, Biodesign Institute, Arizona State University, 1001 S McAllister Ave, Tempe, AZ 85281, USA; svictor4@asu.edu (S.V.); oosoriop@asu.edu (O.O.P.); kevin.abikaram@lau.edu (K.A.K.); aelghoul25@gmail.com (A.E.); madisonransdell@creighton.edu (M.R.); 3 ASU-Mayo Clinic Medical Devices and Methods Laboratory, Health Futures Center, Arizona State University, 6161 E. Mayo Blvd., Phoenix, AZ 85054, USA; 4 School of Engineering for Matter, Transport, and Energy, Arizona State University, Tempe, AZ 85281, USA; 5 School of Computing and Augmented Intelligence, Arizona State University, Tempe, AZ 85281, USA; falhinda@asu.edu (F.A.-H.); teresa.wu@asu.edu (T.W.); 6 Barrow Neurological Institute, 2910 N 3rd Ave, Phoenix, AZ 85013, USA; yonas.geda@commonspirit.org (Y.G.); geetika.chahal@commonspirit.org (G.C.); danielle.eagan@commonspirit.org (D.E.); 7 TF Health Corporation (d.b.a. Breezing Co.), Phoenix, AZ 85054, USA

**Keywords:** Alzheimer’s Disease (AD), Mild Cognitive Impairment (MCI), Alzheimer’s early detection, Smart Driving System, multi-modal sensing array (MMS), cognitive health monitoring

## Abstract

Alzheimer’s disease (AD) and Alzheimer’s Related Dementias (ADRD) are projected to affect 50 million people globally in the coming decades. Clinical research suggests that Mild Cognitive Impairment (MCI), a precursor to dementia, offers a critical window of opportunity for lifestyle interventions to delay or prevent the progression of AD/ADRD. Previous research indicates that lifestyle changes, including increased physical exercise, reduced caloric intake, and mentally stimulating activities, can reduce the risk of MCI. Early detection of MCI is challenging due to subtle and often unnoticed cognitive decline and is traditionally monitored through infrequent clinical tests. In this research, the Smart Driving System, a novel, unobtrusive, and economical technology to detect early stages of neurodegenerative diseases, is presented. The system comprises a multi-modal biosensing array (MMS) and AI algorithms, including driving performance and driver’s biometrics, offering insights into a driver’s cognitive function. This publication is the first work reported towards the ultimate goal of developing the Smart Driving Device and App, integrating it into vehicles, and validating its effectiveness in detecting MCI through comprehensive pilot studies.

## 1. Introduction

Alzheimer’s disease (AD) and AD-related Dementias (ADRD), with their expected rise to affect 13.2 million Americans by 2050, present a significant public health challenge [1,2,3,4]. These conditions are characterized by abnormal changes in the brain that lead to a decline in cognitive function severe enough to disrupt daily life and independence. Symptoms include difficulties in memory, planning, and problem-solving and can profoundly affect behavior, emotions, and relationships. Alzheimer’s Disease, the sixth leading cause of death in the U.S., progressively impairs memory, executive functions, and several other cognitive domains [1].

**Figure 1:**
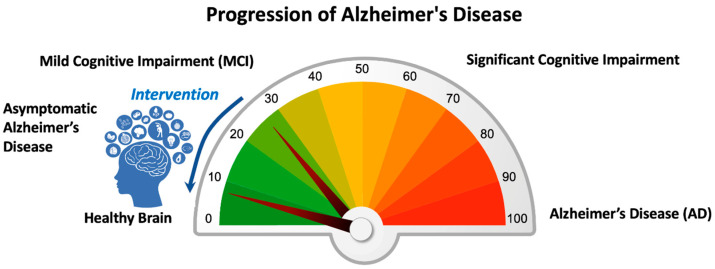
Importance of Early Detection and Intervention of Alzheimer’s Disease and Related Dementias. Schematic representation of Alzheimer’s Disease and Related Dementias Continuum. The scheme represents a hypothesis of reversibility of the brain condition at the early stages of dementia, a.k.a., Mild Cognitive Impairment. This hypothesis is the motivation of the current work to find a method to detect early MCI by incorporating new ways of passive cognitive testing in daily life activities. Note that the scale in the meter is simply for schematic purposes.

AD and ADRD unfold over a continuous time spectrum, beginning with preclinical AD. As AD progresses, individuals transition into the Mild Cognitive Impairment (MCI) phase due to AD, where subtle symptoms like memory and language difficulties begin to emerge; however, patients with MCI maintain activities of daily living, though they may show some subtle changes in instruments of activities of daily living. Not everyone with MCI will develop AD; some with MCI may even revert to normal cognition [2,3]. At this point in time, there is no treatment that cures or reverses AD. At best, the current treatments delay the progression of MCI to dementia [4]. Figure 1 offers a visual representation of this continuum, emphasizing the progression of the disease and potential intervention points, which are crucial for lifestyle changes and treatments to slow or alter the disease’s trajectory.

Lifestyle changes such as increasing physical exercise [5,6], reducing caloric intake [7], and engaging in mentally stimulating activities [8,9] have shown promise in reducing the risk of progressing from MCI to AD. However, the stealthy nature of MCI, often marked by unnoticed cognitive decline and infrequent clinical evaluations, poses a challenge for timely detection and intervention.

Current cognitive tests are infrequent and consist of a single point in time. These approaches inadequately represent the dynamic nature of cognitive functions, which are influenced by a variety of factors such as stress, sleep, and social and emotional stressors [10,11,12,13]. Given the complexity of these factors, current diagnostic tests are insufficient to capture a person’s true cognitive performance. It is important, then, to develop continuous and prolonged measurements of cognitive performance, particularly in free-living conditions. Such an approach would provide a more nuanced and accurate understanding of the progression of cognitive diseases, which would allow for earlier detection and more effective management of conditions like AD/ADRD.

Driving is a complex task that requires extensive neurocognitive engagement [14,15]. It necessitates the integration of multiple cognitive domains, such as attention, memory, executive function, and visuospatial skills, in a dynamic environment [16]. This makes it a practical, noninvasive way to assess brain function and cognitive challenges and potentially a valuable indicator for detecting early signs of cognitive impairment and neurodegenerative diseases. Machine Learning algorithms analyzing driving data present a promising method for the early detection of MCI and dementia. The LongROAD study collected data from 2977 participants through in-vehicle devices and utilized the Random Forest algorithm for classification [17]. This study showcased significant predictive ability, achieving an F1 score of 0.88 by incorporating both demographic and driving data [17]. Research at the University of Toronto (UoT) and Washington University in St. Louis (WashU) has also indicated a strong connection between cognitive decline and driving habits. At UoT, the use of GPS data and machine learning aimed to detect early signs of cognitive decline, with Random Forest classifiers predicting preclinical AD with substantial accuracy [18]. Using solely GPS driving data, an F1 score of 0.82 was achieved, which increased to 0.88 when both GPS driving data and age were combined [18]. The study at WashU investigated how preclinical AD affects driving behaviors in older adults. Over a period of 2.5 years with 20 participants, the findings revealed that those with preclinical AD drove less frequently, covered fewer miles, visited fewer locations, and exhibited a general decline in driving activities compared to those without preclinical AD [19]. It is important to notice that these previous works focused on driving behaviors and have left unexplored the aspects of comprehensive assessments of driving performance, driver’s biometrics, and driver’s environment.

**Figure 2:**
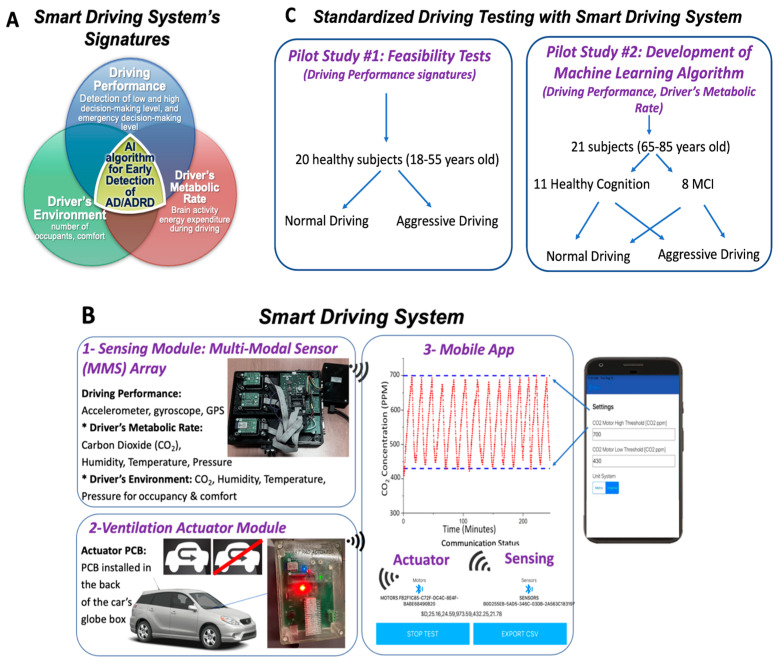
Schematic representation of the: (**A**) Driving signatures captured by the Smart Driving System and their relationship with the artificial intelligence (AI) algorithm for detection of MCI (early AD/ADRD). (**B**) Schematic representation of the Smart Driving System with three wirelessly connected components: (1) Sensing module comprised of a Multi-Modal Sensor Array (MMS array); (2) Ventilation Actuator Module, which has a Printed Circuit Board (PCB) that controls the air recirculation valve inside the car; and (3) Mobile application in a mobile device to assess the MMS array signals and to control the Ventilation Actuator Module. The entire system was placed in a Toyota Matrix, year 2005. (**C**) Schematic representation of the human subjects of the study that participated in Pilot Study #1: Feasibility Tests, and Pilot Study #2: Development of a Machine Learning Model for Assessment of Cognitive Decline.

In the present work, we introduce a new technology for driving assessment named “Smart Driving System”. The system comprises multi-modal sensing (MMS) technology equipped with a comprehensive suite of sensors for the assessment of driving signatures of 1—Driving performance; 2—Driver’s metabolic rate; and 3—Driver’s environment (Figure 2A). 1—Driving performance captures features to assess different decision-making aspects of cognition, such as 90° and 180° turns (low decision-making level), sudden breaks (emergency decision-making level), and path discrepancies from a designated path (high decision-making level). 2—The driver’s metabolic rate captures the energy expenditure of the driver, which is hypothetically related to the driver’s brain activity since the driver does not move significantly while driving. In fact, brain metabolic rate represents 20–25% of the total body metabolic rate at resting state, [20] and age-related metabolic rate decline has been correlated to cognitive functionality [21]. 3—Driver’s environment captures the number of occupants and comfort. In addition, the Smart Driving System’s driving signatures are combined via a machine learning model for the discrimination of healthy cognition and MCI. This is the first step to create an artificial intelligence (AI) algorithm for the detection of MCI (early AD/ADRD).

The Smart Driving System was built in-house (Figure 2B) and tested in a standardized driving environment, using a total of 42 subjects that provided data for our machine learning algorithm training (N = 20 + 21 = 41) and testing (N = 1). The work included (Figure 2C): (1) Pilot study #1, including the system proof-of-concept and feasibility test in 20 healthy subjects (mostly younger adults); (2) Pilot study #2, including the data assessment in 21 older adults to develop and train our machine learning algorithm for discrimination of healthy and MCI subjects, and testing performed in 1 older adult.

## 2. Materials and Methods

### 2.1. Smart Driving System Hardware Overview

Each component of the Smart Driving System includes the following elements and locations inside the car:

*Sensing Module with MMS Array* (Figure 2(B-1)): It has an accelerometer (3-axis accelerometer), gyroscope (3-axis gyroscope), GPS (A-GPS) for dynamic assessment of driving performance metrics, and carbon dioxide (CO_2_, Telaire, General Electrics, New York, NY, USA), temperature (HDC100 Click Mikroe, Belgrado, Serbia), relative humidity (HDC100 Click Mikroe, Belgrado, Serbia), and barometric (Barometer Click, Mikroe, Belgrado, Serbia) sensors for dynamic measurement of driver’s metabolic rate (a.k.a. driving energy expenditure) and driver’s environment (occupancy and comfort). The MMS array is in the rear part of the car. This portion of the array sends the signals to the mobile app via Bluetooth (BLE2 Click, Mikroe, Belgrado, Serbia). Note the accelerometer, gyroscope, and GPS were provided by Danlaw Inc. (Novi, MI, USA) and are the same components included in the data logger described below.

In addition, and as a mode of redundancy sensing, the MMS array includes an on-board diagnostics (OBD) data logger (DCM970, BitBrew Inc. subsidiary of Danlaw, Inc., Novi, MI, USA) (not shown in Figure 2) with additional accelerometry, gyroscope, and GPS, and data sent to the cloud using Amazon Web Services and data collection in the BitBrew platform. Note: This second part of the MMS array was used as an experimental alternative data collection.

*Ventilation Actuator Module* (Figure 2(B-2)): It has a customized printed circuit board (PCB) with Bluetooth (BLE2 Click, Mikroe, Belgrado, Serbia) that interfaces with the vehicle’s air ventilation/conditioning system to manage cabin air quality based on real-time CO_2_ readings from the Mobile App (see below). More specifically, the PCB connects to the air recirculation valve of the car (located on the side of the glove box) and controls the car cabin’s recirculation ventilation when the preset user-selected maximum CO_2_ level inside the car cabin is reached. When CO_2_ levels exceed a maximum set threshold, the system actuates the car’s inlet door to alter air circulation, letting fresh air inside the cabin. This allows the cabin to maintain CO_2_ within good indoor air quality levels since it is known that unhealthy CO_2_ levels can build up quickly inside a car cabin [22]. The PCB is powered by a 12 V source from the car and stepped down to 5 V.

**Figure 3:**
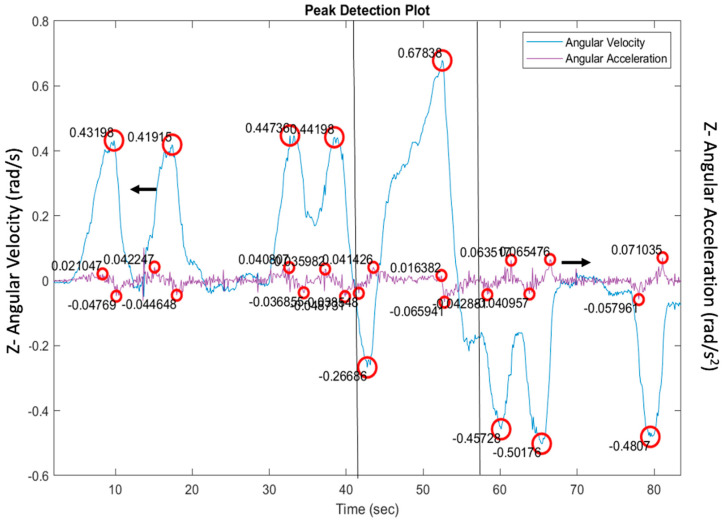
Plot of angular velocity and angular acceleration vs. time with automatic peak detection. The *Y*-axis on the left of the plot is the angular velocity, and the red circles highlight the minimum and maximum values of the angular velocity and the minimum and maximum values of the angular acceleration. The figure represents one lap of the standardized driving test with the black lines separating the type of turn (4 left turns, followed by a U-turn, then 3 right turns).

*Mobile App* (Figure 2(B-3)): It is an iOS application that hosts algorithms with defined functions of sensing, actuation, information processing, and data analytics. It receives, processes, and stores the MMS array sensor signals. In addition, it communicates to the Ventilation Actuator Module via Bluetooth and has a logic defined by user-selected maximum and minimum threshold levels for CO_2_ that triggers the functions of the Actuator Module.

### 2.2. Human Subjects

A total of forty-two volunteers were recruited for the study. All the subjects were educated about the study design and purpose. An informed consent form approved by the Institutional Review Board at Arizona State University (IRB protocol number: STUDY00006547) was obtained from each subject before the study. The study participants included random volunteers or referrals to Arizona State University. All volunteers were treated equally, and their cognitive conditions were neither discussed nor commented on prior to the tests.

### 2.3. Standardized Driving Tests

The subjects followed a standardized test with a rectangular-shaped defined road circuit in our Health Futures Center’s facility. The test consisted of 10 laps of 540 m ea. with straight sections and 4 consecutive left turns (90° left turns), followed by a U-turn (180° turn), and then 3 consecutive right turns (90° right turns).

### 2.4. Signal Processing Algorithms

The Smart Driving System employs a set of algorithms to process the signals captured by the MMS array. The algorithms are currently managed by different software, but they will be integrated in the future. This section outlines the signal processing algorithms employed to convert raw data into structured, analyzable datasets: 1—Driving performance algorithm; 2—Driver’s metabolic rate algorithm; 3—Driver’s environment algorithm.

#### 2.4.1. Driving Performance Algorithm

This algorithm has two sources of data: one data set from the MMS array in the sensing module shown in Figure 2B and another data set from the OBD device captured in the cloud for redundancy. In this process, we used different pieces of software. The data captured from the MMS array was processed using Matlab^®^ 2024b (MathWorks, Natick, MA, USA) [23]. The data captured from the OBD through the Bitbrew 2024 platform, (Danlaw^®^, Novi, MI, USA) platform was processed through Python^TM^ v.3.1.2 (Wilmington, DE, USA). We used Python^TM^ scripts to sort and clean vehicle-specific diagnostics and performance metrics, ensuring data integrity and usability. This data were equivalent to the MMS array data since the sensors’ components were the same (see details Section 2.1), bringing up an alternative source of data (cloud-based configuration) for future development of the system.

The Matlab^®^ programming platform’s procedure for analyzing (1) gyroscope sensor data and (2) GPS sensor data are explained below:

(1) Gyroscope sensor data analysis: Along this line, a critical component of our approach involves analyzing angular velocity data from the gyroscope, particularly in the direction of the *z*-axis, which is vital for detecting vehicle turns based on the MMS array orientation. We employed an advanced peak-detection algorithm that identifies significant peaks in the gyroscope data corresponding to vehicle turns and calculates angular acceleration by differentiating the angular velocity. To refine the analysis of turn dynamics, a window was established around each angular velocity peak. More specifically, we used a “find peak” function from Matlab^®^ in combination with a set of pre-processing steps that involved choosing the right variables. Once the peak was found, then a fixed number of points were considered for data processing before and after the peak. This windowing technique helped determine the maximum and minimum angular acceleration values for each detected turn and provided a detailed examination of each maneuver. The window was assigned systematically. For example, Figure 3 illustrates this analysis by plotting angular velocity (rad/s) and angular acceleration (rad/s^2^) against time, which visually represents the vehicle’s path through sequences of left turns, a U-turn, and subsequent right turns.

For each angular velocity peak, the speed was also obtained from the GPS. Given the different sampling rates of the GPS data (1 Hz) and the gyroscope data (10 Hz), interpolation was necessary to synchronize these data streams. This step is critical for accurately aligning each peak in angular velocity with corresponding speed values from the GPS data, thus enabling a detailed correlation between the dynamics of the turn and the vehicle’s speed. Additionally, minor turns (part of the larger U-turn) were excluded from the overall analysis. This selective focus ensures that the assessment concentrates on relevant driving performance features and reduces noise in the predictive modeling. The adjustments made to the peak-detection algorithm omitted these insignificant turns, ensuring that only meaningful and impactful data are included in the analysis.

(2) GPS sensor data analysis: Moreover, Matlab^®^ scripts were also developed to plot GPS coordinates sequentially, enhancing the qualitative analysis of the driving paths taken by the subjects. The GPS data were used to quantify deviations of the actual driving path created by the driver from the originally designated path presented at the beginning of the standardized driving. Along this line, five different criteria were assessed: U-turns missed, U-turns in different spots (i.e., unplanned turns), consecutive U-turns performed, off-path (deviation from the designated path (including deviations from the track that were at least eight times larger than the GPS error (+/− 1 m), but still within the perimeter of the parking lot), and exited path (exited the perimeter of the designated path). Each criterion was assessed for the number of occurrences and uniquely scored based on assigned thresholds. Details of the features and scoring guide for the features are presented in Supplementary Information (Section S2. Path Deviation Assessment). A final score was given for each subject, which was inserted into the machine learning model data frame.

#### 2.4.2. Driver’s Metabolic Rate Algorithm

**Figure 4:**
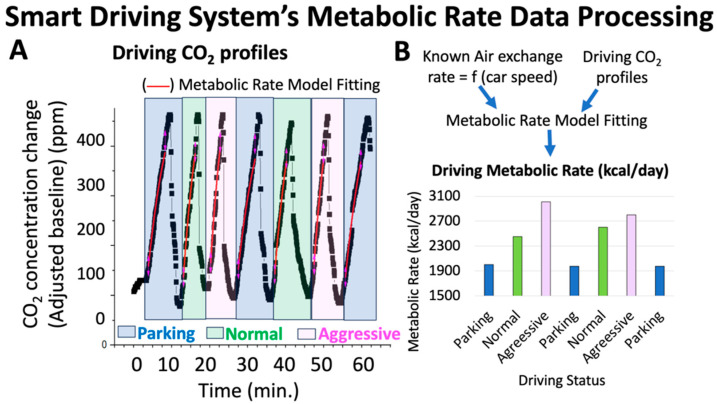
Smart Driving System’s metabolic rate algorithm and representation of the data processing for assessments of the brain activity during different driving conditions: (**A**) CO_2_ profile vs. time in the car cabin due to the presence of the driver recorded during parking, normal driving, and aggressive driving conditions. The portions corresponding to increasing values of CO_2_ are utilized to fit the metabolic rate model represented by Equation (1). (**B**) Top portion: Schematic representation showing the combination of CO_2_ profiles with known air exchange rate inside the car cabin to assess the metabolic rate (Equation (1)). Bottom portion: It shows a representation of the calculated metabolic rate corresponding to the CO_2_-time profiles shown in part A of this figure.

This algorithm is for the extraction of energy expenditure during driving. The data were collected by the mobile app and processed with Origin Lab® 2018 (Northampton, MA, USA). The driver’s metabolic rate, a.k.a. Energy Expenditure (EE), was assessed based on CO_2_ concentration changes recorded within the vehicle cabin (Figure 4).

Figure 4 shows an example of a CO_2_-time pattern extracted from a driver within the car cabin while driving. Utilizing a model developed by the team [22,24,25,26,27,28], and described below, we derive the driving metabolic rate from this pattern. Briefly, we record the accumulation of CO_2_ in the car cabin due to the presence of the driver (Figure 4A). Under this condition, the ventilation recirculation (controlled by the Actuator Module) is on, and the CO_2_ profiles can be described by Equation (1):

(1) $${[CO}_{2}]={{[CO}_{2}]}_{0}+\frac{k_{gen}}{\lambda_{Acc}}\left(1-e^{-\lambda_{Acc}t}\right)+{\left({[CO}_{2}\right]}_{i}-\;{{[CO}_{2}]}_{0})e^{-\lambda_{Acc}t}$$

where CO_2_ has concentration units expressed in parts-per-million (ppm), *λ_Acc_* is the air exchange in the accumulation condition (Acc) with units of hour^−1^, [*CO_2_*]*_i_* is the initial concentration in the CO_2_ accumulation profile, and [*CO_2_*]*_o_* is the outdoor naturally occurring CO_2_ concentration (typically 450 ppm), *k_gen_* is the CO_2_ generation rate in the environment (ppm hour^−1^) due to the driver’s CO_2_ production from the breath. Previous findings [28] suggested that *k_gen_* was correlated to the actual *k_gen_, k_gen_′,* via an environmental correction factor (*CF_env_*) of 1.143, an empirical constant determined to correct for mean error bias originating from any source not accounted for in the “ideal model” used in Equation (1), e.g., imperfect mixing, CO_2_ sensor response time, etc. $k_{gen}^{’}$ is estimated in practice as follows:

(2) $$k_{gen}^{’}=k_{gen}{CF}_{env}$$

Once *λ_Acc_* is estimated by a method described below (see “Estimation of *λ_Acc_*”), *k_gen_* is then assessed for the subject using Equation (1) (Figure 4B) and used to calculate *k_gen_’* from Equation (2). *k_gen_’* is then used in the following equation to determine the value of the driver’s carbon dioxide production rate: *VCO_2_* (mL/min) [27]:

(3) $${VCO}_{2}=k_{gen}’\times V_{Room}\times {CF}_{STPD}/60$$

where *V_Room_* is the car cabin’s volume (ml), and *CF_STPD_* (dimensionless) is a correction factor used to convert measured ambient *VCO_2_* to standard temperature, pressure, and dry conditions (STPD), and it is calculated as follows [27]:

(4) $${CF}_{STPD}=\frac{P_{bar}-P_{H20}}{760}\times \frac{273}{T+273}$$

where *T* represents temperature (Celsius) within the car cabin, which is measured by the SSM array. *P_bar_* (mmHg) is barometric pressure also measured by the SSM array. *P_H20_* is the partial pressure of H_2_O (mmHg) at the car cabin estimated from the measured T, measured relative humidity from the MMS array, and Antoine’s equation. Finally, the driver’s metabolic rate or driving energy expenditure (*EE*) (kcal/day) is calculated using a simplified version of the Weir formula, assuming a constant respiratory quotient (*RQ*) of 0.85 (Figure 4B), according to:

(5) $$EE\left(kcal/day\right)=3.941\times \frac{{VCO}_{2}}{RQ}+1.106\times {VCO}_{2}$$

(6) $$RQ=\frac{{VCO}_{2}}{{VO}_{2}}$$

*Estimation of λ_Acc_:* Since the air exchange rate is hypothetically affected by the car speed, we calibrated the air exchange rate (*λ_Acc_*) as a function of the car speed. As observed from Equation (1), the accurate assessment of metabolic rate depends on the accuracy of the air exchange rate. Therefore, we made the best effort to assess the air exchange rate accurately using the procedure described as follows: Using the k5 (Cosmed, Albano Laziale, Italy) device [29] while driving at conditions mirroring our test setup (e.g., air conditioning level, fan orientation), we measured energy expenditure (*EE*) and *VCO*_2_. We determine the air exchange rate from the measured *VCO*_2_ (*k_gen_*) using Equation (1). Tests were conducted at different speeds to calculate multiple lambda values (*λ_Acc_*), creating a trendline that correlates vehicle speed with the air exchange rate, detailed in Supplementary Information, Figure S1. The formula derived from this trendline is:

(7) $$\lambda Acc=0.2534\times Speed\left(mph\right)+10.323$$

Employing established equations for *VCO*_2_ and energy expenditure (*EE*), we observed significant differences in EE under various driving conditions. As shown in Figure 4 (left), the metabolic rate increased from a parked state to regular driving and peaked during aggressive driving, highlighting the metabolic response to driving intensity and the potential link between metabolic rates and cognitive performance under varying driving conditions. Specifically, heightened driving complexity corresponds to an increased metabolic rate, often by several hundreds of kcal/day. This compelling association suggests that changes in metabolic rate serve as a reliable proxy for assessing brain activity while driving.

It is worth mentioning that the participants performed the three driving phases during the standardized test at parking (no vehicle movement), normal driving (during the standardized test), and aggressive driving (during the standardized test). The participant followed the sequence of parking, normal driving, and aggressive driving in the specified order without any alterations. Due to the cognitively demanding nature of the test and to avoid the potential confounding effects of fatigue, subjects did not repeat the sequence.

#### 2.4.3. Driver’s Metabolic Environment Algorithm

This algorithm determines the number of occupants in the car cabin [22] via CO_2_ production rate. The car occupancy can assist in determining whether the presence of another person in the car influences the driving signatures. This is a distinctive feature that has never been reported before. The mechanism to determine the car occupancy works as follows: Since air quality in the vehicle cabin can be detrimental to cognitive functions [22,30] (>2000 ppm), the Smart Pad System actuates ventilation to maintain safe CO_2_. The algorithm discriminates the presence of additional occupants in the car cabin based on the CO_2_-time profile slope and the times the recirculation/ventilation mode is actuated in a given period of time. The higher the CO_2_–time slope, the more frequent the actuation, the more carbon dioxide generation, and the higher the number of occupants in the car cabin. In the present work, we used a single driver, and therefore, this algorithm was not utilized. However, we introduce this section to illustrate the full capacity of the Smart Driving System.

### 2.5. Development of Machine Learning Models with Customized Feature Engineering

A resampling technique was employed during the training of the model. The dataset was randomly divided into training and testing subsets, and this process was repeated over 100 iterations, each with a unique training/testing split. This approach enhances the robustness of the model and minimizes overfitting, which is particularly important when working with small datasets. The model’s performance was then assessed by averaging the performance across all 100 iterations.

#### 2.5.1. Modeling with Customized Feature Engineering

Based on the data assessed in the pilot studies, we developed: #1—a machine learning algorithm to discriminate between normal and aggressive driving; and #2—a machine learning algorithm to discriminate between normal cognition and MCI. After exploring different machine learning algorithms [31], such as Random Forest, Gradient Boosting Classifier, Support Vector Machine, Logistic Regression, and Gaussian Naïve Bayes, the Random Forest algorithm was selected as it provided the most accurate prediction results with good interpretability.

#### 2.5.2. Random Forest Algorithm

The use of the Random Forest algorithm in this study provided several benefits:

- Handling High Dimensionality: It effectively managed datasets with a high number of features without the need for extensive feature elimination.
- Mitigating Overfitting: By averaging across multiple trees, the algorithm reduced the risk of overfitting, enhancing the model’s generalizability.
- Importance of Features: The model could determine the most significant features in predicting driving performance.
- Versatility: It was suitable for both classification and regression tasks, making it applicable for a wide range of driving data analyses.

This algorithm is a combination of several decision trees. The model builds multiple decision trees and merges them for a more accurate and stable prediction. Each decision tree in a random forest gives a prediction, and the most voted prediction is the algorithm’s output. Every decision tree acts like a “vote” for a class, and then the class with the most votes will be classified [31]. Python® (Wilmington, DE, USA) was used to train and test the model, using the “RandomForestClassifier” model from the “sklearn” library.

The features implemented in the final model include averaged values, resulting in a reduced number of total features. This made the model particularly effective for a dataset that included 20 (pilot study #1) and 21 (pilot study #2) participants, respectively. Our algorithms are available (see Data Availability Statement).

## 3. Results and Discussion

As mentioned in the introduction, we performed two pilot studies with the Smart Driving System: (1) Pilot study #1, which was a feasibility test in 20 healthy subjects (mostly younger adults); (2) Pilot study #2, which was a machine learning model training and testing study, including 21 older adults to develop and model for discrimination of healthy and MCI subjects (Figure 2C); and 1 additional subject for testing the model.

### 3.1. Pilot Study #1: Feasibility Tests

#### 3.1.1. Subjects and Test Characteristics

The initial research phase aimed to establish the Smart Driving System’s ability to differentiate between varied driving performance using a cohort of 20 active community participants aged 18 to 55. The test excluded the CO_2_ analysis and focused on determining key driving performance signatures that were indicative of the quality of driving. Therefore, the participants performed the standardized driving test under two conditions: “normal” driving, without any cognitive perturbation, and “aggressive” driving, where the participants were instructed to drive faster than normal to increase cognitive engagement.

#### 3.1.2. Driving Performance Signature Characterization

**Figure 5:**
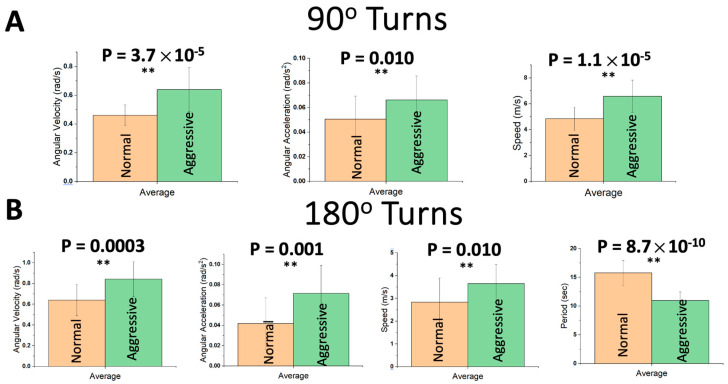
Smart Pad’s driving performance signatures were collected from 20 healthy drivers under normal and aggressive cognitive function conditions. The aggressive cognitive function was induced by instructing the driver to drive aggressively. The figures represent the sensors’ signatures that contribute to the extraction of (**A**) Low-level decision-making events of 90° turns: averaged maximum angular velocity, averaged maximum angular acceleration, and averaged speed at the angular velocity peaks; and (**B**) Low-level decision-making events of 180° turns: averaged maximum angular velocity, averaged maximum angular acceleration, averaged speed at the angular velocity peak, and period (time taken to perform 180° turns). “**” stands for statistically significant difference.

The analysis primarily targeted low-level decision-making events such as 90° and 180° turns. Figure 5 summarizes the driving performance signatures of low-level decision-making events of 90° turns (Figure 5A): averaged maximum angular velocity, averaged maximum angular acceleration, and average speed at the angular velocity peaks; and low-level decision-making events of 180° turns (Figure 5B): averaged maximum angular velocity, averaged maximum angular acceleration, average speed at the angular velocity peak, and period (time taken to perform 180° turns). For the seven driving signatures, statistically significant differences (*p* < 0.05) between different cognitive driving workloads were detected, which indicated that these driving performance signatures are indicative of driving quality.

#### 3.1.3. Driving Performance Signature Machine Learning Model

**Figure 6:**
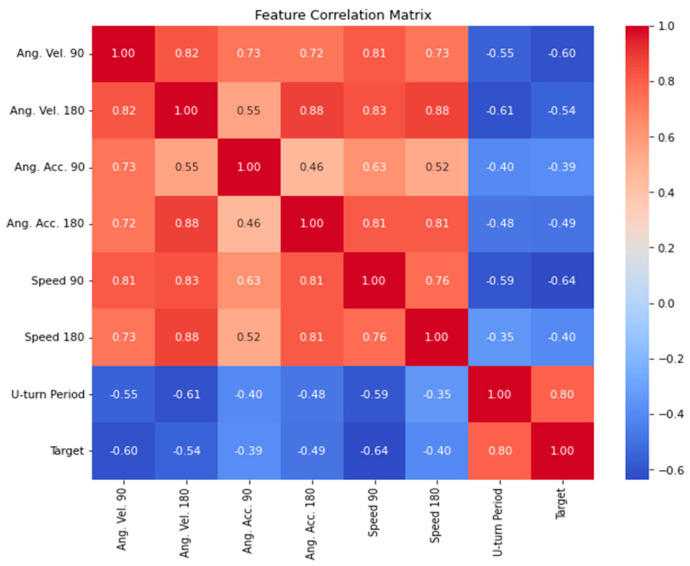
Correlation Matrix for the driving performance signatures. The figure provides an overview of how indicative each driving performance signature is. Ang. Vel. 90 = 90°-turn averaged maximum angular velocity; Ang. Vel. 180 = 180°-turn averaged maximum angular velocity; Ang. Acc. 90 = 90°-turn averaged maximum angular acceleration; Ang. Acc. 180 = 180°-turn averaged maximum angular acceleration. Speed 90 = 90°-turn averaged speed at the angular velocity peaks; Speed 180 = 180°-turn averaged speed at the angular velocity peak; U-turn period = U-180°-turn period (time to perform the 180° turn).

**Figure 7:**
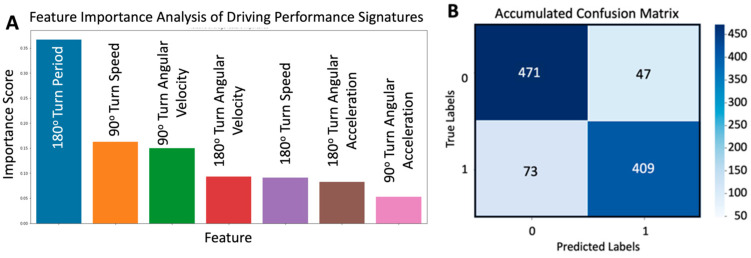
Result of random forest analysis of the Smart Pad’s driving performance signatures from the pilot study with healthy subjects (N = 20): (**A**) Feature importance analysis with the top 7 most relevant features: 1—180° Turn Period = 180°-turn period (time to perform the 180° turn), 2—90° Turn Speed = 90°-turn averaged speed at the angular velocity peaks, 3—90° Turn Angular Velocity = 90°-turn averaged maximum angular velocity, 4—180° Turn Angular Velocity = 180°-turn averaged maximum angular velocity, 5—180° Turn Speed = 180°-turn averaged speed at the angular velocity peak, 6—180 Turn Angular Acceleration = 180°-turn averaged maximum angular acceleration, and 7—90° Turn Angular Acceleration = 90°-turn averaged maximum angular acceleration. (**B**) Accumulated confusion matrix corresponding to driving performance signatures from (**A**) that provided diagnostic accuracy of 88.0% to discriminate normal from aggressive driving with a sensitivity of 84.9%, a specificity of 91.0%, and a precision of 89.7%.

Figure 6 and Figure 7 summarize the data analysis of all the driving performance signatures we evaluated from the standardized tests. We performed an analysis of a total of 16 signatures (90°-left turn averaged maximum angular velocity, 90°-right turn averaged maximum angular velocity, 90°-turn (left + right) averaged maximum angular velocity, 90°-left turn averaged maximum angular acceleration, 90°-right turn averaged maximum angular acceleration, 90°-turn (left + right) averaged maximum angular acceleration, 90°-left turn averaged minimum angular acceleration, 90°-right turn averaged minimum angular acceleration, 90°-turn (left + right) averaged minimum angular acceleration, 90°-left turn averaged speed at angular velocity peaks, 90°-right turn averaged speed at angular velocity peaks, 90°-turn (left + right) averaged speed at angular velocity peaks, 180°-turn period (time to perform the 180° turn), 180°-turn averaged speed at the angular velocity peak, 180°-turn averaged maximum angular velocity, 180°-turn averaged maximum angular acceleration) and concluded that seven of them were the most relevant ones (shown in Figure 6 and Figure 7A). Figure 6 shows a correlation matrix demonstrating the relationships among various signatures (in this case, a target of 1 represents normal behavior while a target of 0 represents aggressive driving behavior), with U-turn duration proving highly significant in behavior prediction. Other features also showed inverse correlations, highlighting their importance in distinguishing driving styles. During model testing, we have concluded that averaging features improved model performance. For instance, rather than separately analyzing maximum and minimum angular velocities for left and right turns, which would create four distinct features, the left and right values were averaged into two consolidated features. This aggregation approach simplified the dataset and slightly enhanced the model accuracy, as models using averaged features performed 2–3% better than those with more granular features.

Finally, we separated the test data with 70% of the data for training and 30% of the data for testing, respectively. Then, we used the Random Forest model and a technique of resampling the dataset (since we had a limited number of samples) and building the model over 100 different training and testing data sets. This process rendered a diagnostic average accuracy of 88.0% to discriminate normal from abnormal (faster) driving with an average sensitivity of 84.9%, an average specificity of 91.0%, and an average precision of 89.7% (Figure 7B), which is a “very good to excellent” level of diagnostic accuracy [32] in discriminating between normal and aggressive driving.

### 3.2. Pilot Study #2: Development of a Machine Learning Model for Assessment of Cognitive Decline

The second pilot study involved 21 subjects aged 65 to 85 (10 females and 11 males). All patients were assessed cognitively through standard-of-care based on published Mayo Clinic criteria [33], which included neuropsychological tests by a behavioral neurologist with expertise in MCI and dementia. Once a diagnosis of MCI was determined, the patient additionally underwent Montreal Cognitive Assessment (MOCA) [34]. MOCA testing was also applied to the other participants from the local Phoenix community. The subjects included 13 normal participants and 8 MCI patients.

#### 3.2.1. Standardized Driving Test Overview

The standardized driving tests were designed to assess participants’ driving performance across three distinct conditions:

*Resting Phase***:** Participants sat in the parked vehicle with controlled air recirculation to maintain CO_2_ levels starting below 600 ppm and increasing to 900 ppm. This phase sets a baseline metabolic rate for comparison to the metabolic rate assessed at the Normal Driving Phase and the Aggressive Driving Phase.

*Normal Driving Phase***:** Participants performed the standardized driving test at normal driving speeds, reflecting a driver’s typical/regular driving behavior in a parking lot in a natural driving environment (average of 8–12 miles per hour).

*Aggressive Driving Phase***:** Participants performed the same standardized driving test at aggressive driving speeds, reflecting an intense driving behavior such as higher driving and turning speeds to simulate more stringent driving conditions (average of 15–21 miles per hour).

#### 3.2.2. Feature Analysis and Extraction

In addition to the 7 features of driving performance (low-level decision-making events) assessed previously in pilot study #1, special attention was paid to the driver’s metabolic rate and high-level decision-making events, which were special signatures expected from the older adult population in this pilot study. More specifically, we extracted two additional features: (1) the driver’s metabolic rate, and (2) path discrepancy, which is a driving performance’s signature of high-level decision-making. For this last parameter, GPS data were analyzed, and path discrepancies were quantitatively assessed to measure how accurately drivers followed the pre-defined path in the course.

**Figure 8:**
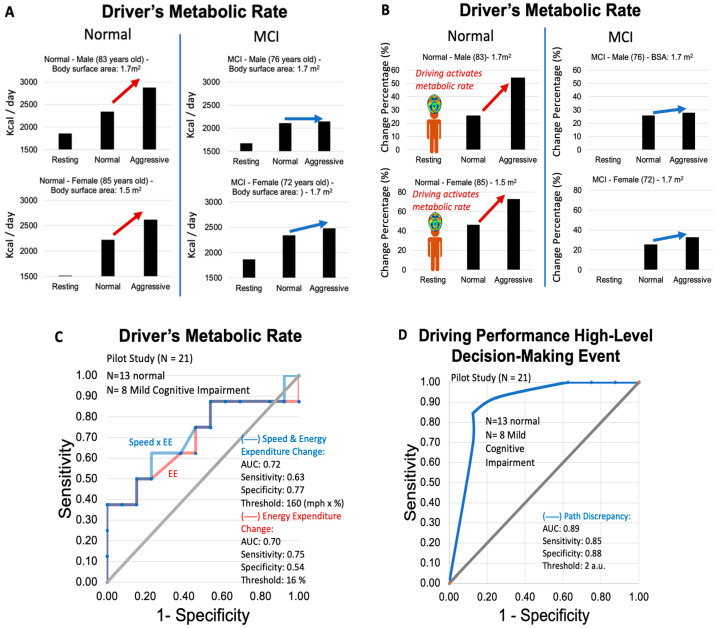
(**A**,**B**) Driver’s metabolic rates during standardized tests for older adult human subjects with normal cognition (left) and MCI (right) conditions: (**A**) Absolute values of drivers’ metabolic rates for stages of parking, normal driving, and aggressive driving; (**B**) the corresponding changes of driver’s metabolic rate from parking to normal driving and parking to aggressive driving. (**C**) Receiving Operating Characteristic curves evaluating the diagnostic accuracy of the driver’s metabolic rate (EE) and driver’s energy expenditure in connection with the car speed (Speed x EE) to discriminate between normal cognition and MCI. (**D**) Receiving Operating Characteristic curves evaluating the diagnostic accuracy of high-level decision-making events (a.k.a. path discrepancy) to discriminate between normal cognition and MCI during the standardized test. Both “driver’s metabolic rate in combination with aggressive driving speed”, and “path discrepancy resulted in a high level of diagnostic accuracy in the older adults’ pilot study (see Section 3.1.3).

*Driver’s Metabolic Rate:* We assessed the driver’s driving metabolic rate according to the procedure explained in Figure 4 in the 21 human subjects. For normal cognition older adults, we observed increasing trends in driving metabolic rates when the drivers transitioned from parking to normal driving to aggressive driving (Figure 8A, left side), with changes in metabolic rate greater than 16% for the drivers going from normal to aggressive (higher speed) driving (Figure 8B, left side). On the contrary, for MCI older adults, we observed significantly smaller increases in driving metabolic rates (Figure 8A, right side), as well as smaller changes in driving metabolic rate from normal to aggressive driving (less than 16% for most cases) (Figure 8B, right side). Therefore, we performed a Receiving Operating Characteristic (ROC) analysis of the driver’s metabolic rate in connection with cognitive status (normal vs. MCI). We determined a diagnostic accuracy of 70% for driving metabolic rate change from normal to aggressive driving (e.g., EE change) with a sensitivity of 75.0%, a specificity of 54.0%, and a diagnostic threshold of 16%. In addition, we found the diagnostic accuracy can be increased to 72.0% if we multiplied the speed by the EE change percentage, resulting in a sensitivity of 63.0%, a specificity of 77.0%, and a threshold of 160 (mph × %). Note mph × % is the aggressive driving speed times the energy expenditure change (%) from normal to aggressive driving (Figure 8C). This metabolic rate-speed hybrid indicator was later optimized for the development of a machine-learning model (see Section 3.1.3).

*Path Discrepancy—Driving performance’s high-level decision-making indicator:* We also assessed high-level decision-making events by evaluating the discrepancies from the path, aka “path discrepancy”, established during the standardized test. Path discrepancy was established with a scale on arbitrary units established from missed 180° turns, 180° turns created due to missed paths, consecutive 180° turns (which were not included in the standardized test path), deviations from the designated path, and exiting earlier from the path. These “path discrepancy” features were not observed in the healthy subjects assessed in the pilot study #1 (healthy, relatively younger adults) and appeared as a strong signature in the human subjects of pilot #2 (with MCI and relatively older adults) with a diagnostic accuracy of 89% according to the receiving operating characteristics curve (Figure 8D).

#### 3.2.3. Development of a New Machine Learning Model for Early Detection of Cognitive Decline: Model Performance and Results

The “Path deviation” indicator was combined with the Speed × Driver’s Metabolic Rate (EE) change shown before. The new indicator feature was as follows:

(8) $$Feature=\frac{{Speed}^{2}\times EEChange}{0.1\times Speed+PathDeviation}$$

**Figure 9:**
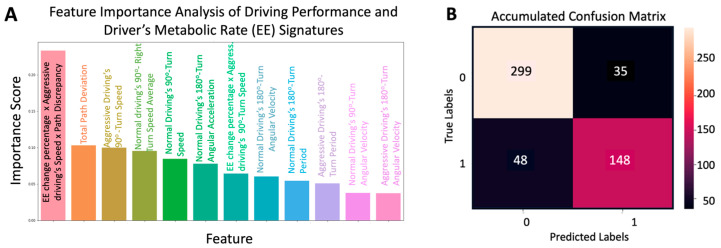
Result of random forest analysis of the Smart Pad’s driving performance signatures from the 2nd pilot study with older adults’ human subjects (N = 21): (**A**) Feature importance analysis with the top 12 most relevant features: 1—EE change percentage x Aggressive driving’s Speed x Path Discrepancy = Equation (8)’s parameter, 2—Total Path Deviation = Scoring (Supplementary Information), 3—Aggressive Driving’s 90^0^-Turn Speed = Aggressive Driving’s 90°-turn averaged speed at the angular velocity peaks, 4—Normal Driving’s 90°-Right Turn Speed Average = Normal Driving’s 90°-right turn averaged speed at the angular velocity peaks, 5—Normal Driving’s 90°-Turn Speed = Normal Driving’s 90°-turn speed at the angular velocity peaks, 6—Normal Driving’s 180°-Turn Angular Acceleration = Normal Driving’s 180°-turn averaged maximum angular acceleration, 7—EE change percentage × Aggressive driving’s 90°-Turn Speed = EE change percentage × Aggressive driving’s 90°-turn speed at the angular velocity peaks, 8—Normal Driving’s 180°-Turn Angular Velocity = Normal Driving’s 180°-turn averaged maximum angular velocity, 9—Normal Driving’s 180°-Turn Period = Normal driving’s 180°-turn (time to perform the 180° turn), 10—Aggressive Driving’s 180°-Turn Period = Aggressive driving’s 180°-turn (time to perform the 180° turn), 11—Normal Driving’s 90°-Turn Angular Velocity = Normal driving’s 90°-turn averaged maximum angular velocity; 12—Aggressive Driving’s 180°-Turn Angular Velocity = Aggressive Driving’s 180°-turn averaged maximum angular velocity. (**B**) Accumulated confusion matrix corresponding to driving performance signatures from (**A**) that provided diagnostic accuracy of accuracy of 87.4%, sensitivity of 85.9, specificity of 82.4, and precision of 89.4%.

We used this indicator feature in our machine-learning model. The optimized model ultimately incorporated a total of 12 features shown in Figure 9A, including 10 features directly obtained from the dataset and 2 engineered features (combination of features): the feature from Equation (8) and the path deviation. This refined feature set was evaluated using a confusion matrix, the results of which are depicted in Figure 9B. Using a similar approach to pilot study #1, the matrix of pilot study #2 resulting from 100 iterations of data resampling rendered an average accuracy of 87.4%, average sensitivity of 85.9%, average specificity of 82.4%, and an average precision of 89.4%.

These metrics demonstrate a marked improvement in model performance over the sole use of Energy Expenditure Change (accuracy = 0.70) or Speed × Energy Expenditure Change (accuracy = 0.72) as shown in Figure 8C, highlighting the benefits of data preprocessing and feature optimization. In addition, we could obtain a high level of diagnostic accuracy without the need to include the driver’s age or any other demographic information. All the findings from the new Smart Driving System seem crucial for achieving reliable and accurate predictions from the model, validating the methods used to refine the dataset, and selecting the most impactful features.

#### 3.2.4. New Machine Learning Model Testing for Early Detection of Cognitive Decline

**Figure 10:**
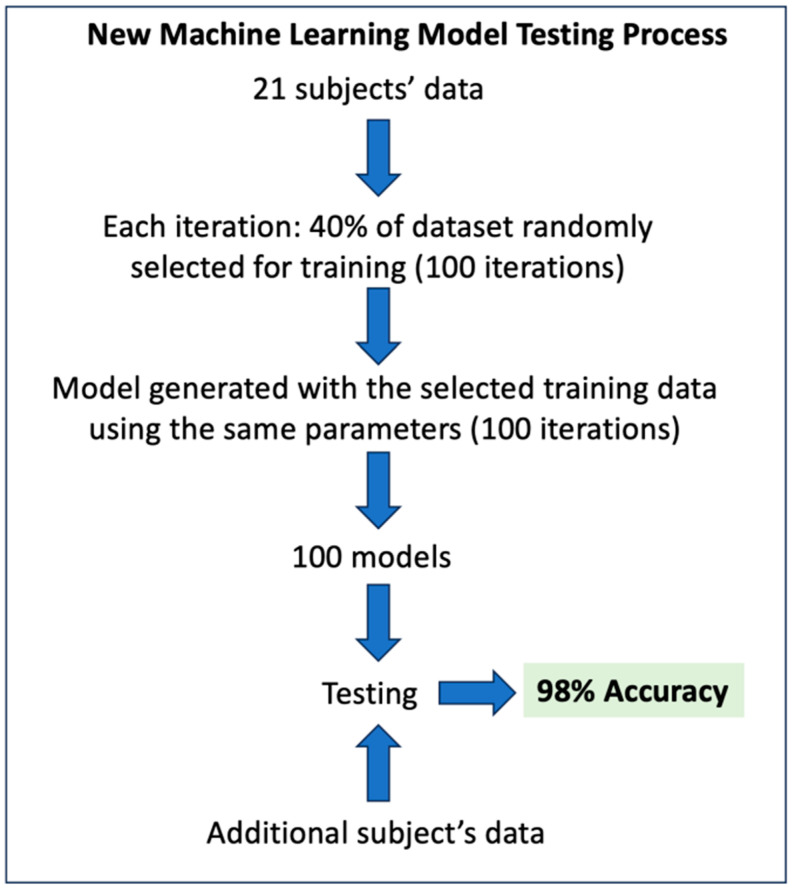
Schematic representation of the process followed for testing (exploratory validation) of the New Machine Learning Model for Early Detection of Cognitive Decline.

The machine learning (ML) model was tested on an additional subject (male, 78 years old, MCI diagnosis) as a method to verify the model’s accuracy when tested with additional data. Figure 10 shows a schematic representation of the testing process. The original data from pilot study #2 containing the 21 subjects was used as a reference. Approximately 100 iterations over the dataset were performed because 1 iteration would simply yield 100%. We used 40% of the dataset as training for the model. For each iteration, the model was trained based on the currently sampled training dataset, and then the model was used to predict the additional subject’s cognitive status. The accuracy achieved over 100 iterations is 98%. In other words, the model was able to correctly predict whether the subject had MCI in 98 out of 100 iterations, which shows the effectiveness of the model and all the used features. The accuracy achieved was higher than the previously obtained averaged accuracy due to non-averaged results and using only this subject’s prediction result as the accuracy, which could have had data that matched the model’s training. It is important to note that the additional subject data represents exploratory validation rather than a definitive one. A more extensive dataset, encompassing a larger cohort of both healthy individuals and those with Mild Cognitive Impairment (MCI), has been planned and is required to perform a rigorous validation of our algorithm.

## 4. Conclusions

The Smart Driving System is a novel approach for the non-invasive detection of cognitive decline related to Alzheimer’s Disease and Related Dementias. By integrating a multimodal biosensing array with an AI algorithm, the system analyzes driving performance and drivers’ metabolic rate responses through CO_2_ analysis to identify early signs of cognitive decline.

The analysis of driving performance indicators for low-level decision-making events such as 90° turns and U-turns revealed that parameters related to angular acceleration and velocity during these maneuvers are significant for modeling driving performance, while features associated with speed and duration during U-turns contributed less. These insights underscore the complexity and importance of using multiple combined driving performance indicators.

In pilot study #1, the system and ML model proved to be effective in determining the type of driving performance in young healthy drivers, while in pilot study #2, the system and ML model were characterized with a diagnostic accuracy of 87% to distinguish individuals with Mild Cognitive Impairment (MCI) from healthy subjects within an older adult group of 21 subjects. This was particularly evident when analyzing changes in metabolic rates alongside deviations from set driving paths. Further, the model was tested in an additional subject and correctly predicted MCI with an accuracy of 98%.

Future efforts will focus on refining the Smart Driving System by optimizing the hardware footprint, improving the software, enlarging the dataset with more diverse driving data, and conducting broader pilot studies.

In summary, this first work marks a significant advancement in the early detection of cognitive decline through innovative monitoring of driving performance and metabolic rates, offering a scalable, cost-effective tool for early diagnosis and management of neurodegenerative diseases. The ongoing development of the Smart Driving System could fundamentally transform our approach to diagnosing and treating the early stages of Alzheimer’s Disease and related conditions.

## Data Availability

For more inquiries concerning data or data processing algorithms, please contact the corresponding authors.

## Supplementary Materials

The following supporting information can be downloaded at: https://www.mdpi.com/article/10.3390/s24248062/s1, Figure S1: Variation of Lambda with Respect to Speed. Table S1: Path Deviation Scoring Method.
