# Supplementary material for: Smart Driving Technology for Non-Invasive Detection of Age-Related Cognitive Decline
Source: Sensors (Basel). 2024 Dec 18;24(24):8062. doi: 10.3390/s24248062 (PMC11679439; doi:10.3390/s24248062)
Supplement: Supplementary file 1 [file sensors-24-08062-s001.zip › sensors-3189152-supplementary.pdf]

sensors

Supplementary Information

Smart Driving Technology for Non-Invasive Detection of Age-Related Cognitive Decline

Peter Serhan [1,2,3], Shaun Victor [2], Oscar Osorio Perez [2,4], Kevin Abi Karam [2,3], Anthony Elghoul [2,3], Madison Ransdell [2], Firas Al-Hindawi [5], Yonas Geda [6], Geetika Chahal [6], Danielle Eagan [6], Teresa Wu [5], Francis Tsow [2,7,*] and Erica Forzani [2,3,4,*]

[1] School of Electrical, Computer and Energy Engineering, Arizona State University, Tempe, AZ 85281, USA; pserhan@asu.edu
[2] Center for Bioelectronics and Biosensors, Biodesign Institute, Arizona State University, 1001 S McAllister Ave, Tempe, AZ 85281, USA; svictor4@asu.edu (S.V.); oosoriop@asu.edu (O.O.P.); kevin.abikaram@lau.edu (K.A.K.); aelghoul25@gmail.com (A.E.); madisonransdell@creighton.edu (M.R.)
[3] ASU-Mayo Clinic Medical Devices and Methods Laboratory, Health Futures Center, Arizona State University, 6161 E. Mayo Blvd., Phoenix, AZ 85054, USA
[4] School of Engineering for Matter, Transport, and Energy, Arizona State University, Tempe, AZ 85281, USA
[5] School of Computing and Augmented Intelligence, Arizona State University, Tempe, AZ 85281, USA; falhinda@asu.edu; falhinda@asu.edu (F.A.-H.); teresa.wu@asu.edu (T.W.)
[6] Barrow Neurological Institute, 2910 N 3rd Ave, Phoenix, AZ 85013, USA; yonas.geda@commonspirit.org (Y.G.); geetika.chahal@commonspirit.org (G.C.); danielle.eagan@commonspirit.org (D.E.)
[7] TF Health Corporation (d.b.a. Breezing Co.), Phoenix, AZ 85054, USA
* Correspondence: frant@breezing.co (F.T.); eforzani@asu.edu (E.F.)

S1. Relationship of Air Exchange Rate and Car Speed

Before calculating the energy expenditure for different driving conditions, it was essential to calibrate the air exchange rate (λ) for our test environment and account for possible cabin air leaks. Using the k5 Cosmed device [6], we conducted analyses to determine the vehicle cabin's air exchange rate under consistent conditions mirroring our test setup (e.g., air conditioning level, fan orientation). This device accurately measured resting energy expenditure (REE) and VCO_2, essential for calibrating an accurate λ model for our vehicle.

Tests were conducted at various speeds to calculate multiple lambda values, creating a trendline that correlates vehicle speed with air exchange rate, detailed in Figure S1.

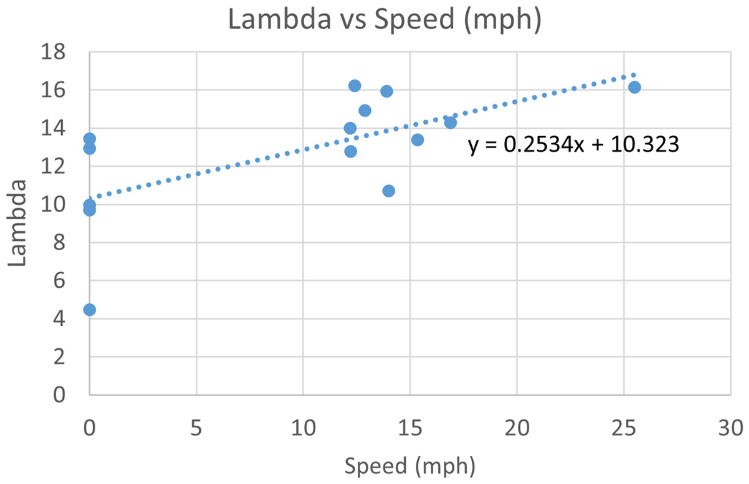

Figure S1. Variation of Lambda with Respect to Speed.

S2. Path Deviation Assessment

An important feature introduced in the analysis of standardized driving tests in the Pilot Study #2 was the assessment of path deviation. This involved analyzing the extent to which a driver's actual path deviated from the predefined loop within the parking lot. By quantifying deviations, the study aimed to gauge the driver's ability to maintain a prescribed route under various driving conditions, offering insights into spatial navigation skills and attention focus. Path deviation was assessed through GPS data, comparing the recorded driving route with the designated path to identify and measure instances of divergence from the path.

Due to the challenge of quantifying path deviation in a confined space such as a parking lot, a combined qualitative and quantitative approach was adopted for inclusion in the machine learning model. This involved plotting the participant's path during both normal and aggressive driving, followed by a qualitative analysis that resulted in a quantifiable score. The deviation from the path was broken down into several components:

- **U-turns Missed:** The number of designated U-turns not performed by the participant.
- **U-turn in Different Spots:** Instances where U-turns were made but not at the designated location.
- **Consecutive U-turns:** More than one U-turn performed consecutively, leading to turns greater than 180 degrees and deviating from the standard U-turn.
- **Off Path:** Situations where the participant veered off the set path but remained within the parking lot.
- **Exited the Parking Lot:** The most significant deviation, occurring when the participant leaves the parking lot area.

This analysis employed a scoring system to quantify deviations from the designated driving path, providing a structured method to assess driving performance more accurately. Each deviation type was scored to reflect the degree of variance from expected behavior, with higher scores indicating greater deviations. This scoring approach effectively translates qualitative observations of driving patterns into quantifiable metrics required for the machine learning model. The criteria for scoring each deviation type are detailed in Table 1. Selection of scoring thresholds was based on the analysis of test results from the participants, ensuring that the scoring method accurately reflects the range and severity of observed driving deviations.

Table S1. Path Deviation Scoring Method.

Scoring Method	U-Turns Missed	U-Turns in Different Spots	Consecutive U-Turns	Off Path	Exited Path
0	≤ 1	≤ 1	0	0	0
1	>1 and ≤ 3	>1 and ≤ 3	>0 and ≤ 2	–	0
2	>3 and ≤ 5	>3 and ≤ 5	>2 and ≤ 4	>0 and ≤ 3	–
3	>5	>5	>4	>3	–
4	–	–	–	–	>0

The scoring thresholds detailed in Table 1 were applied to calculate the path deviation score for each participant. Path deviation emerged as a significant indicator among participants, with an 89% diagnostic accuracy based on the receiver operating characteristic (ROC) curve as illustrated by Figure 8D in the manuscript. This shows the significance in the path deviation feature in determining cognitive decline from driving behavior.
